# Longitudinal trends in PrEP familiarity, attitudes, use and discontinuation among a national probability sample of gay and bisexual men, 2016–2018

**DOI:** 10.1371/journal.pone.0244448

**Published:** 2020-12-31

**Authors:** Ian W. Holloway, Evan A. Krueger, Ilan H. Meyer, Marguerita Lightfoot, David M. Frost, Phillip L. Hammack

**Affiliations:** 1 Department of Social Welfare, UCLA Luskin School of Public Affairs, Los Angeles, California, United States of America; 2 Gay Sexuality and Social Policy Initiative, UCLA Luskin School of Public Affairs, Los Angeles, California, United States of America; 3 Department of Preventive Medicine, USC Keck School of Medicine, Los Angeles, California, United States of America; 4 Williams Institute, UCLA School of Law, Los Angeles, California, United States of America; 5 Department of Medicine, University of California, San Francisco, California, United States of America; 6 Department of Social Science, University College, London, United Kingdom; 7 Department of Psychology, University of California, Santa Cruz, California, United States of America; University of New South Wales, AUSTRALIA

## Abstract

This study explored familiarity with, attitudes toward, uptake and discontinuation of PrEP (Pre-exposure prophylaxis) among a national probability sample of gay and bisexual men. PrEP is one of the most effective biomedical HIV prevention strategies; however, use among gay and bisexual men remains low within the United States. This study used a national probability sample of gay and bisexual men from three age cohorts of men (18–25, 34–41, and 52–59 years at wave 1) who completed three annual surveys between March 2016 and March 2018 (N at wave 1 = 624). Recruitment occurred through a Gallup dual-frame sampling procedure; results for this study came from eligible individuals who consented to be part of the self-administered online or mailed survey questionnaire. We used descriptive data with sampling weights to understand trends in PrEP familiarity, PrEP attitudes and PrEP use across all three time points. Next, PrEP uptake and discontinuation were assessed among men completing all three surveys and who remained eligible for PrEP at all three time points (N = 181). PrEP familiarity increased considerably between 2016 and 2018 among those eligible for PrEP (from 59.8% from wave 1 to 92.0% at wave 3). Favorable attitudes toward PrEP increased more modestly (from 68.3% at wave 1 to 72.7% at wave 3). While PrEP use increased by 90% between the two time points (from 4.1% in 2016 to 7.8% in 2018), this represented a small percentage of overall uptake among eligible participants across time (6.6%). Among respondents who reported PrEP use at wave 1 or wave 2, 33.3% subsequently discontinued PrEP use at a later wave. Findings indicate modest increases in PrEP use between 2016 and 2018 in a national probability sample of sexually-active gay and bisexual men. PrEP discontinuation was high and suggests the need for further research into gay and bisexual men’s PrEP discontinuation and persistence.

## Introduction

Pre-exposure prophylaxis (PrEP) is an established biomedical HIV prevention strategy to reduce incident infections among gay, bisexual and other men who have sex with men (GBMSM). PrEP involves daily use of one of two HIV medications, Truvada^®^ or Descovy^®^, to prevent HIV acquisition [1,2], and is a principal strategy of the United States’ “Ending the HIV Epidemic: A Plan for America” [3]. Since 2012, when daily dosing of Truvada^®^ for PrEP was first approved by the Food and Drug Administration, familiarity with this prevention strategy has increased considerably, with estimates indicating an increase in familiarity from 47.4% in 2013 to 80.6% in 2017 among a national non-probability sample of PrEP‐eligible MSM in the U.S. [4]. In recent years new PrEP dosing strategies (e.g., intermittent or on-demand) have been approved, with additional PrEP modalities, such as injectable PrEP and topical PrEP, in the pipeline [5].

Attitudes toward PrEP among GBMSM have been mixed since Truvada^®^ was first approved in 2012 [6]. Stigma surrounding the use of PrEP [7], concerns regarding long-term side effects [8], conflicting messages from AIDS service providers [9], and lawsuits and social media misinformation campaigns [10] have possibly created confusion about PrEP’s benefits. A national probability sample of sexually active gay and bisexual men estimated over two-thirds (68.4%) of those familiar with PrEP held favorable attitudes toward the prevention strategy in 2016 [11]. No national data derived from probability samples on PrEP attitudes have been published since that time.

Despite stigma and confusion regarding the benefits of PrEP, studies suggest high levels of willingness to take PrEP among GBMSM [12,13]. For example, a 2020 study of young GBMSM (ages 13–18) demonstrated over two-thirds of study participants had previously heard about PrEP, and over 90% reported willingness to take PrEP, to prevent HIV [13]. PrEP uptake among GBMSM has been slow within the United States [14,15]. Data from Gilead, the pharmaceutical manufacturer of Truvada^®^ and Descovy^®^, estimates approximately 140,000 Truvada^®^ prescriptions filled in 2018. This number is far short from the estimated 1.2 million people in the United States that are at substantial risk of acquiring HIV [8]. Further complicating our understanding of PrEP uptake is the lack of national probability samples of GBMSM. The only study to date using a national probability sample of sexually-active gay and bisexual men estimated PrEP coverage at approximately 4% [11].

Once oral daily PrEP initiation occurs, safe and effective use depends on adherence to the medication [16,17] and persistence on PrEP in the context of HIV risk behavior. Adherence challenges related to structural barriers (e.g., insurance coverage) [18] and individual-level challenges (e.g., side-effects) [19] contribute to reduced protection against HIV infection among GBMSM [20,21]. Several studies have noted that adherence challenges may also result in PrEP discontinuation. However, discontinuation may also occur in the context of reduced HIV risk (e.g., monogamous relationships), the decision to enact other HIV prevention strategies (e.g., consistent condom use), or the experience of significant side effects such as bone density loss and/or misinformation about side effects [10,22]. To our knowledge, no data from national probability samples have been published on PrEP persistence among gay and bisexual men.

This study explored longitudinal trends in familiarity with, attitudes toward, uptake and discontinuation of PrEP among sexually-active gay and bisexual men using a U.S. national probability sample. Studies derived from probability samples have the advantage of generalizability (i.e., should be representative of the population of interest).

## Materials and methods

The current study examined data from male participants in the *Generations Study*, which used a national probability sample of lesbian, gay, and bisexual (LGB) adults in three age cohorts recruited from all 50 U.S. states and the District of Columbia. The study methods are described in detail elsewhere [23]. Three distinct age cohorts (ages 18–25, 34–41, and 52–59 at screening) were targeted that represent different historical periods which contextualized the experience of LGB adults in the U.S.

A national probability sample was collected by Gallup using a dual-frame sampling procedure, with random-digit dialing of both landlines and cell phones [24]. Respondents were interviewed using personal phone interviews that determined whether they identified as lesbian, gay, bisexual, or transgender (LGBT). Those responding affirmatively were then further assessed for eligibility for a second survey, for which respondents were eligible if they (a) identified as gay, lesbian, bisexual, queer, or same-gender loving, (b) were not transgender, (c) were Black, Latino, or White race/ethnicity, (d) were ages 18–25, 34–41, or 52–59 years old, (e) completed a sixth-grade education, and (f) answered the phone interview in English. Transgender respondents were invited to participate in a concurrent study focused on the experiences of transgender people. In the second step, eligible respondents who consented to be part of the survey self-administered a comprehensive online or mailed survey questionnaire. The results reported here were collected in the second-step survey.

In total, 366,644 participants were screened by Gallup between March 2016 and March 2017. Of them, 12,837 (3.5%) identified as LGB, 3,525 met eligibility criteria, 2,882 (82%) agreed to participate, and 1,345 (47%) completed the survey, for a total conditional participation rate of 38%. Participants responded to the survey by self-administering the study questionnaire either online via a link provided in an email or on paper via a mailed questionnaire returned in a pre-stamped, pre-addressed envelope. Participants read an information sheet prior to beginning the survey and consented by completing the questions and submitting it to the researchers. Gallup (the company that conducted the survey) collected identifying information in order to maintain contact with respondents over time. The researchers did not have access to any personal identifying information of participants, who were compensated $25 for participating in the survey.

Participants were followed from wave 1 for two additional waves of yearly data collection. Wave 1 data have been published previously [11], and occurred between March 2016 and March 2017. Wave 2 data collection occurred between April 2017 and March 2018. Wave 3 of data collection occurred between April 2018 and March 2019. The present analyses were limited to respondents who identified as male (wave 1 N = 624, 40.5% of the sample). Eligibility for PrEP was determined separately at each wave. To be considered as eligible for PrEP, respondents were required to report having sex with another man in the past 5 years (at wave 1) or the past 1 year (waves 2 or 3), and to be HIV negative. Accounting for attrition, 470 men were eligible for PrEP at wave 1 (75.2%), 285 were eligible at wave 2 (66.7%), and 223 were eligible at wave 3 (70.5%). There were 181 men who completed all three waves of data collection and were PrEP eligible at all three waves. These procedures were approved by the Institutional Review Board of the University of California, Los Angeles and collaborating institutions.

## Measures

### PrEP variables

PrEP familiarity was assessed with the question, “Truvada is a pill that HIV-negative people can take to prevent HIV infection. This is called PrEP (or Pre-Exposure Prophylaxis). How familiar are you with Truvada as PrEP?” Responses were dichotomized: “Not at all familiar” vs. “Familiar” (“somewhat familiar,” “very familiar”). Data were collected prior to Descovy^®^’s approval as PrEP.

PrEP attitudes were assessed with the question, “Are you for or against HIV-negative people taking Truvada as PrEP to prevent the transmission of HIV?” Responses were dichotomized: “For it” (“I am for it”) vs. “not for it” (“I am against it,” “I have mixed feelings about it,” “I don’t have an opinion,” “I don’t know enough about it”).

PrEP use was measured with the question, “Are you currently taking Truvada as PrEP?” (yes/no). Responses to this question at each wave were also used to measure (1) uptake (i.e., participants who reported not using PrEP at wave 1 but reported using at wave 2 or wave 3 and participants who reported not using at wave 2 but reported using at wave 3); (2) discontinuation (i.e., participants who reported using PrEP at wave 1 or wave 2 and not at wave 2 or wave 3); and (3) persistence (i.e., participants who reported using PrEP at all 3 waves).

### Demographic variables

Membership in three age cohorts (18–25, 34–41, 52–59) were assigned based on date of birth.

Sexual identity was self-reported. Responses were collapsed into three categories: gay, bisexual, other, including, for example, queer, pansexual, and same-gender loving.

Education was dichotomized (high school or less: “less than a high school diploma,” “high school graduate”; more than high school: “technical, trade, vocational, or business school or program after high school,” “some college,” “two-year associate degree,” “four-year bachelor’s degree from a college or university,” “some postgraduate or professional schooling after graduating college,” “postgraduate or professional schooling after graduating college”).

Race/ethnicity was assessed at the phone interview screening. Respondents were categorized into single-race categories as follows: Respondents who answered “yes” to the question, “Are you of Hispanic, Latino, or Spanish origin–such as Mexican, Puerto Rican, Cuban, or other Spanish origin?” were categorized as Latino/Hispanic, regardless of other races endorsed. Respondents who endorsed “Black or African American” were categorized as African American/Black, regardless of other races endorsed (with the exception of those categorized as Latino/Hispanic). Respondents were categorized as White if they endorsed White race, including any additional race except for those endorsing African American/Black and those who were categorized as Latino/Hispanic. Eligibility was limited to these racial groups (or multiple racial and ethnic identities that included at least one of these) because estimates showed that we would not be able to recruit a sufficient number of respondents who were Asian (5.9% of U.S. population) or Native American/Alaskan Native (1.3%) to satisfy power requirements for Generations [24].

Urbanicity scores were calculated using respondents’ residential zip codes according to the USDA Rural-Urban Commuting Area (RUCA) coding system [25]. RUCA scores of 1–3 represented urban zip codes, while scores greater than 3 represented non-urban zip codes.

## Data analysis

Data were analyzed descriptively across all three waves. In a first set of analyses, PrEP familiarity, attitudes and use were assessed cross-sectionally at each wave among men who were PrEP eligible (N = 470 men at wave 1; N = 285 at wave 2; and N = 223 at wave 3). Next, among respondents who completed all three waves and who were PrEP eligible across all three waves (N = 181), we assessed PrEP uptake, discontinuation and persistence. All analyses were weighted, allowing for generalization to the U.S. population of gay and bisexual men from each of the three age cohorts under study. Weights were developed by Gallup and adjusted for nonresponse bias.

## Results

Table 1 presents descriptive statistics by wave of data collection among PrEP eligible respondents.

**Table 1:** Demographics of a national probability sample of gay and bisexual men presented cross-sectionally, 2016–2018^1^.

	Wave 1		Wave 2		Wave 3	
	N = 470		N = 285		N = 223	
	%_w_	95% CI	% _w_	95% CI	% _w_	95% CI
Cohort						
*Younger*	52.89	47.42, 58.29	53.18	45.54, 60.67	57.86	48.73, 66.48
*Middle*	24.87	20.45, 29.88	27.54	21.18, 34.95	25.14	17.93, 34.03
*Older*	22.24	18.67, 26.27	19.28	14.98, 24.47	17.00	12.51, 22.69
Sexual Identity						
*Gay*	73.57	68.21, 78.31	76.73	69.22, 82.86	80.40	71.64, 86.95
*Bisexual*	22.26	17.72, 27.58	17.13	11.56, 24.64	16.00	9.84, 24.94
*Other*	4.17	2.717, 6.358	6.14	3.733, 9.924	3.60	1.95, 6.55
Race/ethnicity						
*White*	65.96	60.2, 71.28	64.69	56.37, 72.2	62.70	52.44, 71.93
*Black/African-American*	12.56	8.86, 17.50	12.90	7.74, 20.72	15.65	8.923, 25.99
*Latino/Hispanic*	21.49	17.08, 26.67	22.42	16.24, 30.09	21.65	14.36, 31.28
Education						
*More than High School*	61.61	55.52, 67.36	59.02	50.47, 67.06	53.66	43.84, 63.20
Urbanicity						
*Urban*	89.56	85.76, 92.43	92.22	87.99, 95.04	95.82	91.55, 97.98
*Non-Urban*	10.44	7.57, 14.24	7.78	4.96, 12.01	4.18	2.02, 8.45
Census Region						
*Northeast*	19.39	15.54, 23.91	19.43	14.25, 25.91	18.79	12.71, 26.89
*Midwest*	20.91	16.63, 25.95	23.66	17.61, 31.01	22.20	15.42, 30.87
*South*	33.12	28.03, 38.64	31.18	24.23, 39.10	37.45	28.55, 47.29
*West*	26.58	22.12, 31.59	25.73	19.85, 32.65	21.56	15.23, 29.59

Table reports raw sample sizes and weighted percentages.

^1^Among participants eligible for PrEP at each respective wave.

The majority of PrEP eligible participants identified as gay (wave 1: 73.57%; wave 2: 76.73%; wave 3: 80.40%) and the majority identified as white (wave 1: 65.96%; wave 2: 64.69; wave 3: 62.70%). Over half had received more than a high school education (wave 1: 61.61%; wave 2: 59.02%; wave 3: 53.66%) and the vast majority lived in urban settings (wave 1: 89.56%; wave 2: 92.22%; wave 3: 95.82%).

**Fig 1:**
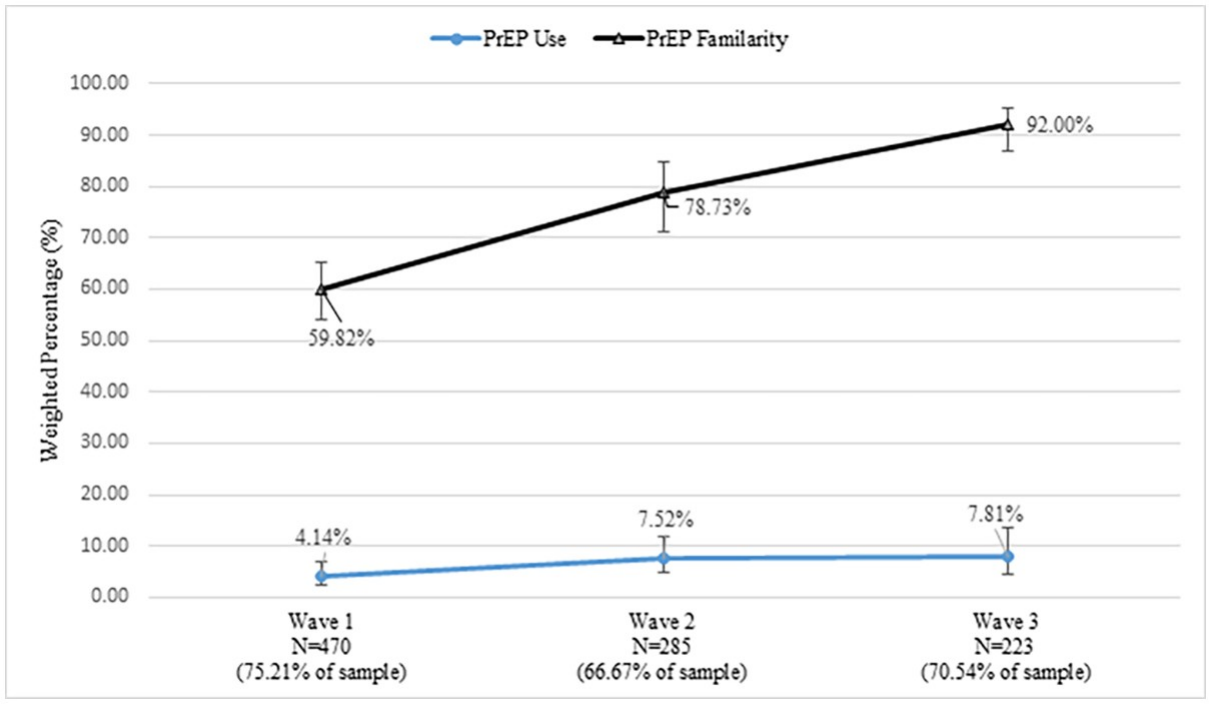
PrEP familiarity and use among a national probability sample of gay and bisexual men presented cross-sectionally 2016–2018*. *N = respondents determined eligible for PrEP at that specific wave.

Fig 1 presents PrEP familiarity and use across the three study time points among those eligible at each wave. At wave 1, nearly two-thirds of participants (59.82%) were familiar with PrEP. This increased at wave 2 (78.73%) and increased again at wave 3 (92%), a 54% increase from wave 1 to wave 3. PrEP uptake also increased across all three waves: In wave 1, 4.14% of eligible participants used PrEP; by wave 3, 7.81% of those eligible used PrEP, an almost 90% increase.

**Fig 2:**
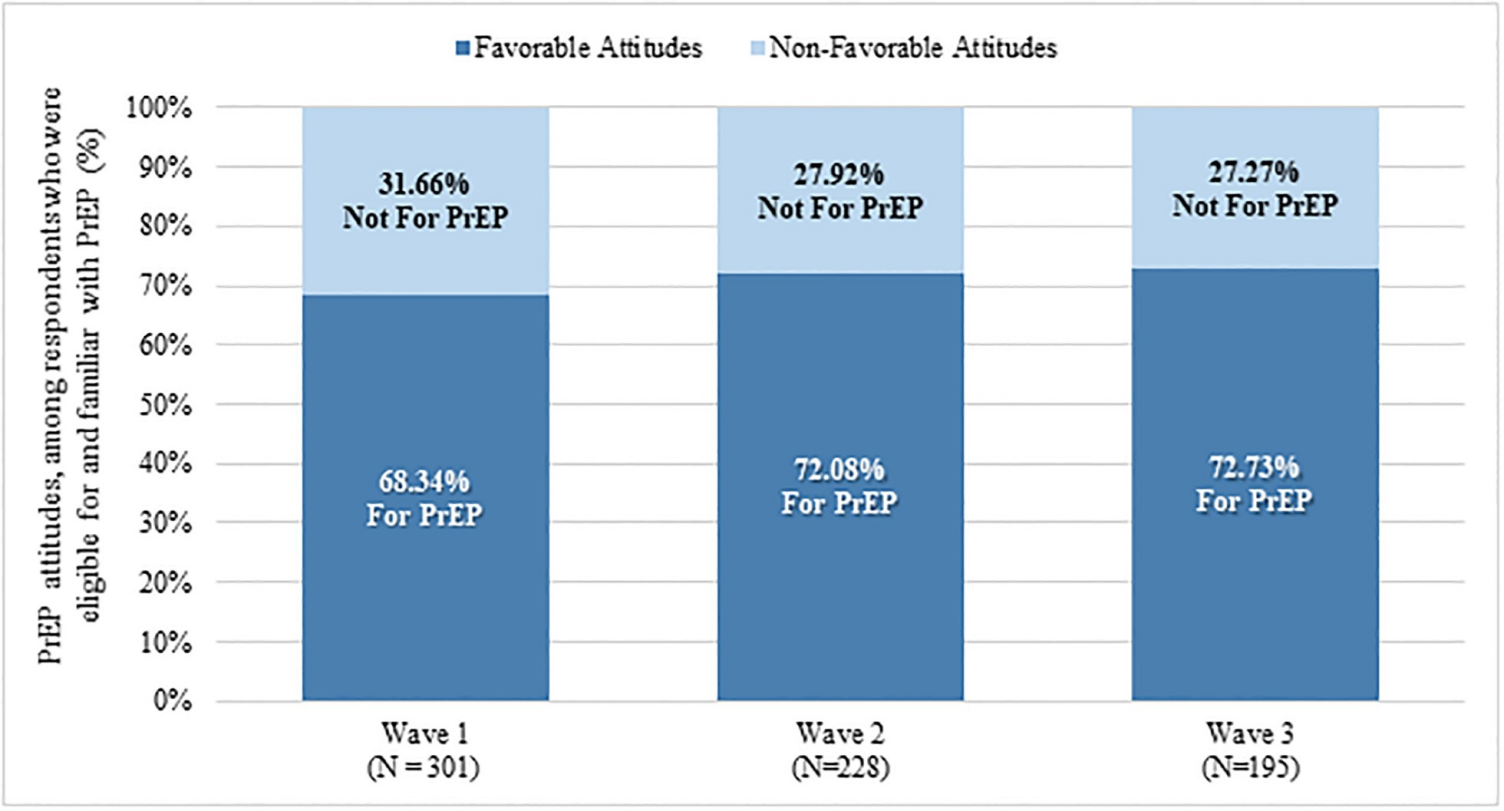
Attitudes toward PrEP among a national probability sample of gay and bisexual men presented cross-sectionally, 2016–2018*. *N = Respondents determined eligible for and familiar with PrEP at each wave.

Fig 2 represents PrEP attitudes among those eligible for PrEP and familiar with PrEP at each wave of data collection. Attitudes of being “for PrEP” increased from wave 1 to wave 2 (68.34% to 72.08%) but remained relatively stable between wave 2 and wave 3 (72.08% to 72.73%).

**Fig 3:**
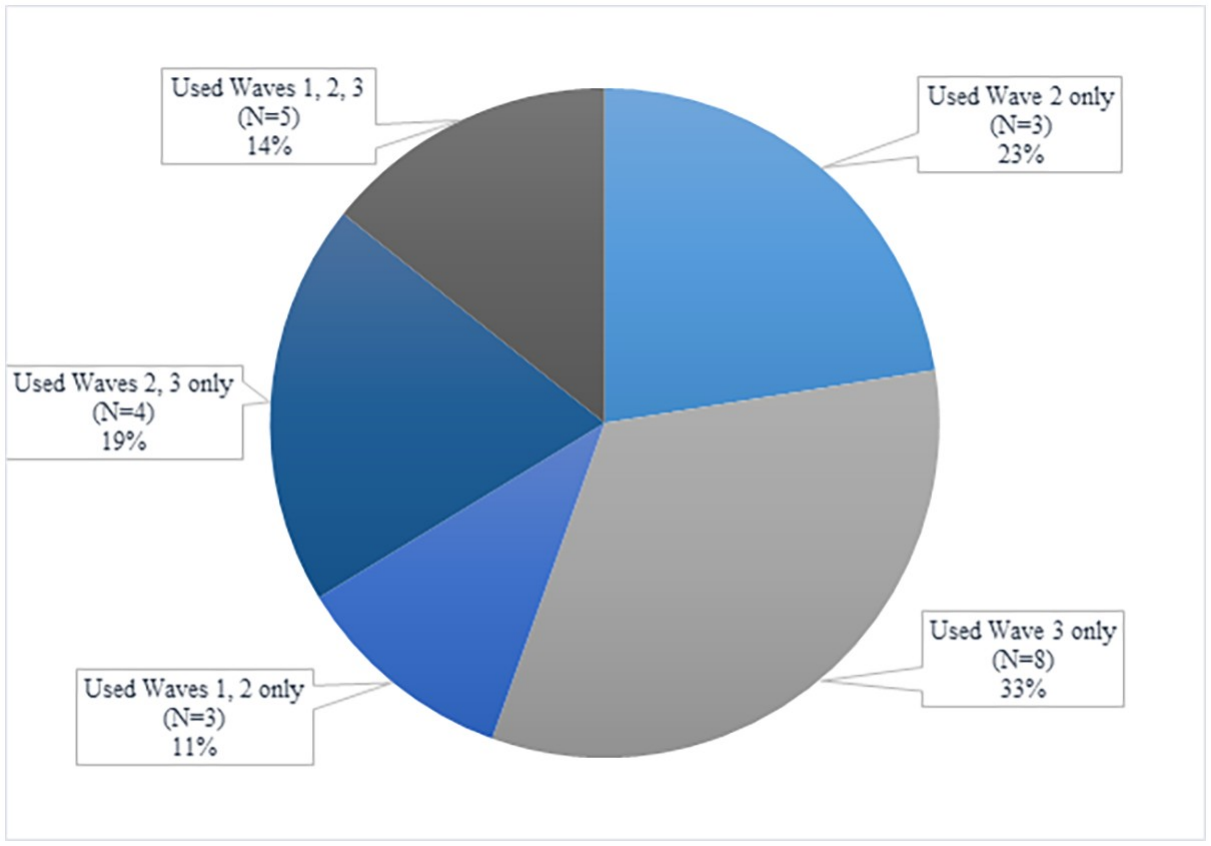
PrEP uptake, discontinuation and persistence among a national probability sample of gay and bisexual men presented cross-sectionally, 2016–2018 (N = 23*). *While 24 participants reported ever using PrEP at any wave, 1 respondent was missing a PrEP use response at wave 1. With this missing information, the waves at which they used PrEP could not be accurately categorized, and so they are excluded from Fig 3. The individual’s valid PrEP use responses at waves 2 (“no”) and 3 (“yes”) are included in overall uptake calculations.

PrEP trajectories across PrEP users are presented in Fig 3. Among those who were eligible for PrEP across all waves (N = 181), 13.3% used PrEP at *any* wave (n = 24). There was 6.6% uptake over any of the three waves, and 33.3% discontinuation over any of the three waves (one participant was missing a valid PrEP use response at wave 1, reported no PrEP use at wave 2, but did report PrEP use at Wave 3. Given their response pattern, we were able to include them in the calculation for PrEP uptake but were unable to assess whether they discontinued from PrEP).

Of those who used PrEP at any wave (and had complete PrEP use information at all waves, n = 23), a small percentage (11%) remained on PrEP between waves 1 and 2 but not in wave 3 (discontinuation), less than a quarter (n = 3, 23%) used PrEP at wave 2 only (both uptake and discontinuation), a third (n = 8, 33%) used at wave 3 only (uptake), and 19% (n = 4) used at both waves 2 and 3, but not 1 (uptake). Fourteen percent used consistently *across all waves* (persistence).

## Discussion

Our findings, through this national probability sample of gay and bisexual men, support existing research on widespread PrEP familiarity, and comparatively low PrEP use. Attitudes toward PrEP were mostly positive (“for it”), but more than a quarter of gay and bisexual men who were both eligible for and familiar with PrEP in our study indicated a negative (“not for”) PrEP attitude at each wave. PrEP use across multiple waves of data (persistence) was low and 33% of those who used PrEP discontinued use over the course of the study.

Familiarity with PrEP was high and increased over time. These data are largely consistent with other studies [26–28] and demonstrate the success of current education efforts. Favorable attitudes toward PrEP were high across all three waves but increased only slightly over time. Furthermore, more than a quarter of men were not for PrEP and this remained stable between the second and third data collection points. These data may indicate both the success of national PrEP awareness campaigns (e.g., prepster.info) and the need for improved education about PrEP and its benefits. Prior studies have demonstrated the important association between PrEP knowledge, attitudes and willingness to use PrEP [29–31]. PrEP stigma, fear about side effects, and PrEP lawsuits may contribute to unfavorable attitudes among gay and bisexual men [10,32,33]. Further research is warranted to understand and address negative attitudes toward PrEP.

Our data reinforce that PrEP use among gay and bisexual men remains low in the United States [34]. Our national probability estimate of 6.6% PrEP uptake over the course of our study is on the low end of estimates for PrEP uptake from clinical [35–37]; and community samples [14,38,39]. Although our data demonstrate PrEP use increased by 90% (from 4.14% to 7.81%) from wave 1 to wave 3, indicating greater coverage over the course of three years, there is substantial room for improvement.

A number of barriers to PrEP uptake have been identified, including cost, insurance coverage, lack of access to a provider, poor patient-provider communication, concerns about side effects, and stigma [7,33,40–43]. Structural challenges must be addressed through policy change and scholars have suggested nationwide Medicaid expansion and generic formulations of PrEP to address cost concerns [13,44]. Some states, like California and New York, have implemented successful PrEP assistance programs that cover medication as well as ancillary costs (e.g., lab testing) [45,46]. Models outlining the PrEP continuum of care (also called the PrEP cascade) demonstrate the need for accurate and reliable PrEP information dissemination to support informed PrEP uptake [47–49].

PrEP discontinuation in our sample was high (33%), which mirrors data from clinical settings, where PrEP discontinuation after six months ranges from 37–62% [50–53]. While we do not have data to understand reasons for discontinuation, previous research suggests both structural and individual level barriers to and facilitators of PrEP persistence. Commonly cited structural factors contributing to discontinuing PrEP include difficulty attending routine medical appointments [18,54], lack of insurance coverage [55,56] and high cost of the medication [57]. At the individual level, GBMSM have cited side-effects [58], other adherence challenges [59] and changes in HIV risk behavior as key reasons for PrEP discontinuation [60]. More research on motivations for discontinuation is warranted [61].

## Limitations

Study findings should be interpreted in light of some limitations. The data were collected via self-report and may be subject to social desirability and/or recall bias. We used sex with a man in the past 5 years (wave 1) and 1 year (follow-up) as a proxy for PrEP eligibility, which is less stringent than the clinical recommendations for PrEP put forward by the CDC [62]. The small number of PrEP users prevented us from running analyses on correlates of PrEP use, discontinuation and persistence. This includes differences by race/ethnicity and age cohort, two important factors in ongoing efforts to increase PrEP uptake among gay and bisexual men [63]. Future research with larger samples should focus on age cohort and racial/ethnic differences in PrEP engagement. Because these data were part of a larger study to understand a variety of health and well-being indicators among LGB people, no sexual behavior data or data on PrEP dosing strategies was collected. This prevents us from understanding PrEP uptake and discontinuation in context of behavioral risk. In addition, we limited the racial/ethnic groups because estimates showed that we would not be able to recruit a sufficient number of respondents who were Asian (5.9% of U.S. population) or American Indian/Alaskan Native ([AI/AN], 1.3%) to satisfy power requirements for the *Generations Study*. We encourage future research with Asian and AI/AN communities, which are not included in our sample. Familiarity with PrEP may have been influenced by participation in the study and questions regarding PrEP at multiple waves. As noted above, we did not collect data on reasons for PrEP discontinuation, which is an area for future research with national probability samples of gay and bisexual men.

## Public health implications

Our research has important public health implications for PrEP implementation with gay and bisexual men. First, a substantial portion of participants endorsing unfavorable attitudes toward PrEP indicate the need for more widespread education and accurate information about the benefits of PrEP [10]. Low PrEP use in our national probability sample also indicates room for improvement in strategies designed to make PrEP more accessible. While PrEP discontinuation in and of itself is not a negative outcome, especially in the context of calculated risk assessment, prior research indicates many structural challenges to both PrEP uptake and persistence. Multi-level interventions to improve PrEP accessibility among gay and bisexual men are warranted.

## Conclusions

Our study is the first to document longitudinal trends in PrEP use among a national probability sample of gay and bisexual men at risk for HIV infection. Findings point to steep increases in PrEP familiarity from 2016–2018 with more modest gains in attitudes “for PrEP” and PrEP uptake. In addition, we found high rates of PrEP discontinuation over this time period. Future research with national probability samples of gay and bisexual men should be conducted to understand structural- and individual-level factors that contribute to PrEP persistence and/or discontinuation among gay and bisexual men across the country with a focus on regional differences. These data are needed to inform national recommendations that could lead to more robust healthcare policy to support PrEP access, uptake, and persistence in this population disproportionately impacted by HIV.
